# Comprehensive histopathological analysis of gastric cancer in European and Latin America populations reveals differences in PDL1, HER2, p53 and MUC6 expression

**DOI:** 10.1007/s10120-024-01578-3

**Published:** 2025-01-05

**Authors:** Carolina Martínez-Ciarpaglini, Rita Barros, Carmelo Caballero, Hugo Boggino, Lorena Alarcón-Molero, Bárbara Peleteiro, Erika Ruiz-García, Edith Fernandez-Figueroa, Roberto Herrera-Goepfert, Consuelo Díaz-Romero, Rui Ferreira, Tessa S. Groen-van Schooten, Cinthia Gauna, Rita Pereira, Daniel Cantero, Horacio Lezcano, Federico Esteso, Juan O´Connor, Arnoldo Riquelme, Gareth I. Owen, Marcelo Garrido, Juan Carlos Roa, Fiorella Ruiz-Pace, Ana Vivancos, Marc Diez-García, Maria Alsina, Judit Matito, Agatha Martin, Marina Gómez, Ester Castillo, Maria Vila, João Santos-Antunes, Andreia Costa, Florian Lordick, Judith Farrés, Brenda Palomar-De Lucas, Manuel Cabeza-Segura, Rosanna Villagrasa, Elena Jimenez-Martí, Ana Miralles-Marco, Rodrigo Dienstmann, Sarah Derks, Ceu Figueiredo, Andrés Cervantes, Fátima Carneiro, Tania Fleitas-Kanonnikoff

**Affiliations:** 1 Department of Pathology, Hospital Clinico Universitario, INCLIVA, Biomedical Research Institute, University of Valencia, Valencia, Spain; 2 Ipatimup, Institute of Molecular Pathology and Immunology of the University of Porto, Rua Júlio Amaral de Carvalho 45, 4200-135 Porto, Portugal; 3 i3S-Instituto de Investigação e Inovação em Saúde, Universidade Do Porto, Porto, Portugal; 4 Faculty of Medicine of the University of Porto, Porto, Portugal; 5 Department of Pathology, Unidade Local de Saúde São João, Porto, Portugal; 6 Department of Pathology, GENPAT, Asunción, Paraguay; 7 Department of Pathology, Hospital General de Valdepeñas, Valdepeñas, Spain; 8 Hospital Epidemiology Center, University Hospital Center of São João, Porto, Portugal; 9 Department of Public Health and Forensic Sciences, and Medical Education, Faculty of Medicine, University of Porto, Porto, Portugal; 10 EPIUnit-Institute of Public Health, University of Porto, Porto, Portugal; 11 Laboratory for Integrative and Translational Research in Population Health (ITR), University of Porto, Porto, Portugal; 12 Departamento de Tumores de Tubo Digestivo, Instituto Nacional de Cancerología, Mexico City, México; 13 Laboratorio de Medicina Traslacional, Instituto Nacional de Cancerología, Mexico City, México; 14 Núcleo B de Innovación en Medicina de Precisión, Instituto Nacional de Medicina Genómica, Mexico City, México; 15 Departamento de Patología Quirúrgica, Instituto Nacional de Cancerología, Mexico City, México; 16 Departamento de Oncología Médica, Instituto Nacional de Cancerología, Mexico City, México; 17 Microbes & Cancer. i3S, Instituto de Investigação e Inovação em Saúde, , Rua Alfredo Allen, 208, 4200-135 Porto, Portugal; 18 Department of Medical Oncology, Amsterdam University Medical Center (UMC) Location Vrije Universiteit Amsterdam, Amsterdam, Netherlands; 19 Cancer Biology and Immunology, Cancer Center Amsterdam, Amsterdam, Netherlands; 20 Oncode Institute, Amsterdam, The Netherlands; 21 Medical Oncology Department, Instituto de Previsión Social, Asunción, Paraguay; 22 Department of Gastroenterology, Instituto de Previsión Social, Asunción, Paraguay; 23 Pathology Department, Instituto de Previsión Social, Asunción, Paraguay; 24 Medical Oncology Department, Instituto Alexander Fleming, Buenos Aires, Argentina; 25 Department of Gastroenterology, Faculty of MedicineCenter for Prevention and Control of Cancer (CECAN), Pontificia Universidad Catolica de Chile, Santiago, Chile; 26 Faculty of Biological Sciences & Faculty of Medicine, Millennium Institute for Immunology and ImmunotherapyCenter for Prevention and Control of Cancer (CECAN), Advance Center for Chronic Disease (ACCDIS), Pontificia Universidad Católica de Chile, Santiago, Chile; 27 Centro de Oncología de Precisión, Universidad Mayor, Santiago, Chile; 28 Department of Pathology. Faculty of Medicine. Pontificia, Universidad Católica de Chile Santiago, Santiago, Chile; 29 Oncology Data Science, Valld`Hebron Institute of Oncology, Barcelona, Spain; 30 Cancer Genomics Lab, Valld`Hebron Institute of Oncology, Barcelona, Spain; 31 Medical Oncology Department, Valld`Hebron Institute of Oncology, Barcelona, Spain; 32 Hospital Universitario de Navarra, Navarrabiomed-IdiSNA, Pamplona, Spain; 33 Department of Gastroenterology, Unidade Local de Saúde São João, Porto, Portugal; 34 Department of Oncology, Unidade Local de Saúde São João, Porto, Portugal; 35 Department of Medicine (Oncology, Gastroenterology, Hepatology, and Pulmonology), Comprehensive Cancer Center Central Germany (CCCG), University of Leipzig Medical Center, Leipzig, Germany; 36 Anaxomics Biotech, S.L. Barcelona, Barcelona, Spain; 37 Department of Medical Oncology, Hospital Clinico Universitario, INCLIVA, Biomedical Research Institute, University of Valencia, Avenida Menendez Pelayo nro 4 accesorio, Valencia, Spain; 38 Department of Gastroenterology, Hospital Clínico Universitario de Valencia, Valencia, Spain; 39 CiberOnc. Carlos III Institute, Madrid, Spain

**Keywords:** Gastric cancer, Europe, LATAM, *Helicobacter pylori*, Biomarkers

## Abstract

**Introduction:**

Gastric cancer (GC) burden is currently evolving with regional differences associated with complex behavioural, environmental, and genetic risk factors. The LEGACy study is a Horizon 2020-funded multi-institutional research project conducted prospectively to provide comprehensive data on the tumour biological characteristics of gastroesophageal cancer from European and LATAM countries.

**Material and methods:**

Treatment-naïve advanced gastroesophageal adenocarcinoma patients were prospectively recruited in seven European and LATAM countries. Formalin-fixed paraffin-embedded primary tumour endoscopic biopsy samples were collected and submitted for central morphological and immunohistochemical characterization and TP53 molecular assessment and *Helicobacter pylori* infection.

**Results:**

A total of 259 patients were included in the study: 137 (53%) from LATAM and 122 (47%) from Europe. Significant biological differences were detected between European and LATAM patients. Low representation of chromosomal instability (CIN) and HER2 positive cases were found in LATAM. MUC6 and PD-L1 were more frequently overexpressed in European cases, showing a significant correlation across the entire study population, with this association being especially pronounced in MMRdeficient cases. Both TP53 mutation by next-generation sequencing and p53 immunohistochemical aberrant pattern were linked with features associated with chromosomal instability. No regional differences were observed in H. pylori prevalence or abundance, indicating that the afore mentioned variations cannot be attributed to this factor.

**Conclusion:**

Our findings underscore a need for region-specific approaches in gastroesophageal cancer diagnosis and treatment. MUC6 emerges as a putative immune regulator that needs further investigation. Research tailored to the unique biological profiles in different global regions is crucial to effectively address the observed disparities.

**Supplementary Information:**

The online version contains supplementary material available at 10.1007/s10120-024-01578-3.

## Introduction

Gastric cancer (GC) is a major health challenge, being the fifth most diagnosed cancer worldwide and the fifth leading cause of cancer-related deaths globally [1]. Chronic infection by *Helicobacter pylori* (*H. pylori*) is the main risk factor for developing GC and its eradication may be the most cost-effective prevention strategy [2]. Although almost half of the world’s population is infected, the prevalence of *H. pylori* infection varies across continents and is linked to socioeconomic status [3, 4]. However, among *H. pylori*-positive individuals, the burden of GC is significantly different. East Asia, Latin America, and Eastern Europe, merge as high-risk regions associated with the highest prevalence of malignant transformation after acquisition of the infection [4]. These observations underscore the importance of other behavioral, environmental, and genetic risk factors in the development of GC [5, 6]. The epidemiology of GC is dynamic, with a current trend for reduction in global incidence, yet an increase in the proportion of cases in the population under 50 years old. The latter trend is more notable in countries with a low prevalence of *H. pylori* infection [7]. These epidemiological changes and the lack of consistent records for GC prevalence in Latin America (LATAM), veil an accurate assessment of the situation and highlight the need for contemporary data [8].

GC is complex and heterogeneous and is frequently diagnosed at a late stage [9]. Over the past half-century, the histologic classification of gastric adenocarcinoma has been largely based on Lauren’s criteria and the World Health Organization (WHO) classifications [10, 11]. The incidence of GC subtypes within these classifications differs among geographic regions. Non-cardia GC is the predominant subtype globally and through East and Central Asia and Eastern Europe. Conversely, cardia GC is more common in Western countries [12]. Chemotherapy based on a platinum-fluoropyrimidine schemes is the standard therapy for advanced disease. Targeted therapies such as anti-HER2 and immunotherapy have improved the outcomes in combination with chemotherapy for specific subgroups [13, 14]. Immunohistochemistry (IHC) for HER2, programmed death ligand 1 (PD-L1), and mismatch repair (MMR) proteins is extensively used to analyses tumor characteristics that are critical for treatment decisions [15]. However, most GC epidemiological and molecular studies are provided from Asian and European/North American cohorts, and specific regional considerations are not currently accounted for in other settings. Given this situation, the LEGACy study, a Horizon 2020-funded multi-institutional research project was conducted prospectively, recruiting GC samples from European and LATAM countries for a period of 5 years (2019–2023) to evaluate histopathological differences and *H. pylori* infection distribution in both regions [16]. The main goal of this study is to provide knowledge on the biological characteristics of GC in a European and LATAM cohort to assess the presence of regional differences that may offer insights into future tailored therapies and thus improved quality of life for GC patients.

## Methods

### Patient materials

Patient materials were collected as part of an IRB-approved trial called LEGACy (ClinicalTrials.gov Identifier: NCT04015466, July 11, 2019) and local Ethics Committees of each recruiting site approved within the project. The patients were recruited between 2019 and 2023 in four hospitals in three Western EU countries (Spain, Portugal, and the Netherlands) and four hospitals from LATAM countries (Argentina, Mexico, Chile, and Paraguay). Tumor biopsies were prospectively collected from treatment-naïve advanced gastric adenocarcinoma patients, including gastroesophageal junction (GEJ) tumors (AEG II and AEG III according to Siewert and Stein) [17]. After obtaining informed consent, biopsies of the primary tumor were collected during endoscopy. Snap-frozen and formalin-fixed paraffin-embedded (FFPE) samples were used for the different studies. Data collection and handling of patient material was standardized via a uniform Lab handbook. The study protocol required the collection of at least eight tumor fragments through endoscopy to ensure comprehensive sampling and address tumor heterogeneity. To ensure consistency in sample handling across all participating centers, the laboratory handbook provided meticulous details on pre-fixation and fixation procedures. All FFPE and fresh frozen tissues were centrally managed at Instituto de Patologia e Imunologia Molecular da Universidade do Porto (Ipatimup), Portugal. FFPE tissue was subjected to pathological examination and immunohistochemistry and then sent to the Vall d' Hebron Institute of Oncology (VHIO), Spain for DNA analysis. Fresh frozen samples were centrally processed at Ipatimup, Portugal for microbiome analysis.

### Immunohistochemistry and in situ hybridization

FFPE samples were cut in 3-µm sections and stained with Ventana® BenchMark ULTRA (Roche) system together with the OptiView DAB IHC Detection Kit (#760–700, Roche) following standard protocol for the following proteins: PD-L1 (Anti-PDL1 Clone 22C3, Dako), CD3 (NCL-L-CD3-565 Clone LN10, Leica), CD8 (#790–4460 Clone SP57, Roche), FoxP3 (#12,653 Clone D6O8R, Cell Signaling), MSH2 (#790–5093 Clone G219-1129, Roche), MSH6 (#790–5092 Clone SP93, Roche), MLH1 (#790–5091 Clone M1, Roche), PMS2 (#790–5094 Clone A16-4, Roche), MUC6 (#760–4390 Clone MRQ-20, Roche), Ki-67 (#790–4286 Clone 30–9, Roche), TP53 (#790–2912 Clone DO-7, Roche) and HER2 (#790–2991 Clone 4B5, Roche). For all antibodies, antigen retrieval was carried out with CC1 (EDTA) and counterstained with hematoxylin for 8 min. A positively staining control tissue was present on each processed slide. For in situ hybridization (ISH) the Dual ISH DNA Probe Cocktail (800–4422, Roche) and Epstein–Barr Virus (EBV) Early RNA (EBER) (800–2842, Roche) probes were used for HER2 and EBV, respectively, together with the UltraView SISH DNP Detection Kit (#800–098, Roche) and UltraView Red ISH DIG Detection Kit (#800–505, Roche).

### Morphological evaluation, immunohistochemistry and in situ hybridization interpretation

The histological classification was performed in all cases according to both Lauren`s classification and the 5th edition of the WHO classification of Tumours of the Digestive System [10, 18]. Cases presenting tubular morphology were classified as low grade (well and moderately differentiated) or high grade (poorly differentiated), the latter presenting solid structures and barely recognized tubules [18]. We recorded, in all cases, the presence or absence of signet ring cells (SRC) and the proportion of SRC relative to all tumor cells independently on the histological type. SRCs were defined as tumor cells with ample cytoplasmic mucin optically clear on hematoxylin and eosin (H&E) staining with an eccentrically placed nucleus [19].

Cases were classified as MMR proficient (MMRp) if any intensity of nuclear staining of MMR proteins, MLH1, MSH6, MSH2 and PMS2 was present in all neoplastic cells. Peritumoral lymphocytes, stromal cells, and non-neoplastic epithelial cells were used as internal protein controls. Only cases showing loss of nuclear staining with positive internal control for at least one of the proteins were classified as MMR deficient (MMRd).

In the *ISH* study for EBER, the cases showing nuclear staining in 100% tumor cells were considered as EBV-positive [20, 21].

For HER2 analysis, the cases were classified as positive (3 +), equivocal (2 +), or negative (1 + or 0) according to the intensity and extent of the membranous expression of the protein in the invasive adenocarcinoma component following the College of American Pathologists guideline [22]. Cases classified as HER2 equivocal (2 +) were further evaluated by ISH to assess HER2 amplification status.

The percentage of tumor cells showing nuclear p53 expression with any intensity was recorded. Further, a semi-quantitative evaluation of p53 expression by the Histoscore (H-score) was performed. First, we estimated the percentage of tumor cells presenting no staining (score 0), weak (score 1), moderate (score 2), or strong staining (score 3). The H-score for each case was determined by multiplying the score of staining intensity by its corresponding percentage and summing the resulting values, following the formula: [0 × percentage of immunonegative tumor cells] + [1 × percentage of weakly stained tumor cells] + [2 × percentage of moderately stained tumor cells] + [3 × percentage of strongly stained tumor cells]. The resulting H-score ranged in value from 0 to 300. Cases were classified using a dichotomous approach: If the H-score was equal to 0 or greater than 280, cases were classified as having a p53 aberrant pattern [20]. The remaining cases with an H-score between 1 and 279 were considered to have a p53 normal pattern.

The combined positive score (CPS) for PD-L1 was evaluated following the PD-L1 IHC 22C3 pharmDx Interpretation Manual for gastric cancer [23]. The number of positive mononuclear inflammatory cells (cytoplasmic or membranous staining) and positive tumor cells (presenting membranous staining) in the tumor invasive area were recorded separately.

Tumor infiltrating CD3, CD8, and FoxP3 positive lymphocytes were counted using the positive cell detection script of the open-source software Qupath [24]. For CD3 and CD8 counting, two hotspot areas of 0.30 mm^2^ were selected. For FoxP3 counting, one hotspot area of 0.20 mm^2^ was selected. Normal mucosa, gastritis, granulation tissue, and necrosis were avoided. For Ki-67 expression, at least 500 consecutive neoplastic tumor cells were manually annotated in Qupath. The percentage of nuclear positivity was calculated by using the positive cell detection script on the annotated area.

For MUC6, the percentage of positive tumor cells presenting cytoplasmic staining with any intensity was estimated in each case. Normal mucosa, gastritis, intestinal metaplasia, and necrosis were not considered. The cases were divided between MUC6 high or neg/low using 20% of positive cells as cut-off.

### TP53 mutational analysis

Tumor FFPE samples were also used for custom broad NGS (Panel300v4) using Maxwell® 16 FFPE Tissue LEV DNA Purification Kit (Promega), SureSelect XT Library Prep Kit ILM (Agilent Technologies) followed by sequencing in the Illumina MiSeq instrument and in-house bioinformatics pipeline (VarScan2, GATK, frequent SNPs in the population are filtered with the 1000 g database MAF > 0.005) and manual interpretation of variant pathogenicity. The assay covers mutations and copy number alterations in 425 cancer genes.

### Molecular classification

We classified each case into one molecular subtype: EBV-positive, microsatellite instability (MSI), genomically stable (GS), and chromosomal instability (CIN), following an immunohistochemical and ISH algorithm recently proposed [20]. For this, EBER-positive cases were classified as EBV-positive, and MMR protein loss was indicative of MSI. The remaining EBV-negative and MMRp cases were categorized as CIN if p53 expression exhibited an aberrant pattern. If the immunohistochemical pattern of p53 was normal in these MMRp and EBV-negative tumors, the cases were classified as GS.

### *Helicobacter pylori*

The abundance of the *Helicobacter genus* was obtained from amplicon sequence variants (ASVs) and transformed by applying the log10(*x* + 1). ASVs were generated from microbiome sequencing reads analyzed as previously described [25].

### Statistical analysis

All analyses were carried out by using Stata® IC 15.1 (Stata Corp, College Station, Texas, USA). Continuous data were summarized as mean ± standard deviation if the variables were normally distributed or median and interquartile ranges if the variables did not follow a normal distribution. The normality of continuous variables was assessed using the Shapiro–Wilk test. In the case of continuous variables that follow a normal distribution, the two groups were compared using Student’s *t*-test and, for those who did not follow a normal distribution, we used the Mann–Whitney *U* test or Kruskal–Wallis test, as appropriate. Categorical data were presented as counts and proportions and were compared using Pearson χ2.A *p*-value ≤ 0.05 was considered as statistically significant.

## Results

A total of 259 patients with locally advanced and metastatic gastric/GEJ cancer were included in the study: 137 (53%) from LATAM and 122 (47%) from Europe (Table 1).

**Table 1:** Clinicopathological variables in European *vs* Latin American (LATAM) countries

	Total, *n* = 259	LATAM, *n* = 137 (53%)	Europe, *n* = 122 (47%)	*p* value
Laurén Classification				
Intestinal	124 (48%)	59 (43%)	65 (53%)	*0.387*
Diffuse	77 (30%)	43 (32%)	34 (28%)	
Mixed	24 (9%)	14 (10%)	10 (8%)	
Unclassifiable	34 (13%)	21 (15%)	13 (11%)	
WHO classification				
Tubular	115 (44%)	54 (40%)	61 (51%)	*0.357*
Papillary	6 (2%)	5 (3%)	1 (1%)	
Poorly cohesive	77 (30%)	43 (31%)	34 (28%)	
Mucinous	12 (5%)	9 (7%)	3 (2%)	
Mixed	22 (9%)	12 (9%)	10 (8%)	
Hepatoid	1 (1%)	0 (0%)	1 (1%)	
Lymphoid stroma-rich	7 (3%)	4 (3%)	3 (2%)	
Undifferentiated	18 (7%)	10 (7%)	8 (7%)	
Tumor grade^a^				
Low grade	82 (62%)	34 (49%)	48 (77%)	***0****.****001***
High grade	50 (38%)	36 (51%)	14 (23%)	
Tumor location				
Lower	61 (38%)	23 (24%)	38 (34%)	***0****.****015***
Middle	80 (59%)	47 (49%)	33 (29%)	
Upper	67 (39%)	26 (27%)	41 (37%)	
Signet ring cell content				
Mean (mean SD)	11.9 (26.1)	15.1 (29.43)	8.3 (21.45)	*0.055*
Median (min–Max)	0 (5–100)	0 (10–100)	0 (5–100)	
MMR proteins				
MMRp	226 (88%)	123 (90%)	103 (85%)	*0.191*
MMRd	31 (12%)	13 (10%)	18 (15%)	
EBV infection				
Negative	245 (95%)	127 (93%)	118 (97%)	*0.221*
Positive	13 (5%)	9 (7%)	4 (3%)	
HER2				
Negative	236 (93%)	130 (96%)	106 (89%)	***0****.****048***
Positive	19 (7%)	6 (4%)	13 (11%)	
P53 IHC *pattern*				***0****.****001***
Normal pattern	164 (65%)	99 (74%)	65 (54%)	
Aberrant pattern	89 (35%)	34 (26%)	55 (46%)	
TP53 genomic *status* ^b^				*0.235*
Wild-type	92 (55%)	41 (60%)	51 (51%)	
Mutated	76 (45%)	27 (40%)	49 (49%)	
Molecular type				
MSI	31 (12%)	18 (10%)	13 (15%)	** < *0****.****001***
EBV	13 (5%)	9 (7%)	4 (3%)	
GS	129 (51%)	83 (62%)	46 (40%)	
CIN	80 (32%)	28 (21%)	52 (43%)	
PD-L1 CPS				
< 1%	47 (20%)	40 (31%)	7 (6%)	** < *0****.****001***
> = 1%	191 (80%)	88 (69%)	103 (94%)	
< 5%	89 (37%)	66 (52%)	23 (21%)	** < *0****.****001***
> = 5%	149 (63%)	62 (48%)	87 (79%)	
< 10%	118 (50%)	77 (60%)	41 (37%)	** < *0****.****001***
> = 10%	120 (50.%)	51 (40%)	69 (63%)	
CD3				
Mean (mean SD)	1042.9 (938.3)	983.1 (906.4)	1111.4 (973.0)	*0.293*
Median (min–Max)	795 (11–6025)	729 (11–5413)	842 (142–6025)	*0.202*
CD8				
Mean (mean SD)	733.5 (787.9)	687.8 (644.4)	784.9 (923.2)	*0.340*
Median (min–Max)	503.5 (5–7069)	482 (32–2994)	527.5 (5–7069)	*0.594*
FoxP3				
Mean (mean SD)	92.5 (93.8)	63.6 (70.2)	124.5 (105.9)	** < *0****.****001***
Median (Min–Max)	69 (0.4–564)	43 (0.4–288)	89 (7–564)	** < *0****.****001***
MUC6				
Mean (mean SD)	18.6 (29.4)	12.4 (25.2)	25.6 (32.1)	** < *0****.****001***
Median (Min–Max)	0.6 (0–100)	0 (0–100)	10 (0–100)	** < *0****.****001***
Ki-67				
Mean (mean SD)	72.3 (23.8)	69.0 (25.6)	75.8 (21.3)	***0****.****022***
Median (Min–Max)	80 (4–100)	77.0 (4–100)	83.5 (10–100)	***0****.****043***

Values in bold indicate statistically significant differences (*p* < 0.05), while italicized values represent non-significant trends

Staining interpretation in some cases was hindered by lack of tumor representation

^a^Applies to tubular and papillary carcinomas and not to other GC subtypes

^b^Evaluated in a subset of cases

In both regions, the most common histological subtype was intestinal/tubular adenocarcinoma (Lauren and WHO classification, respectively), comprising 43% and 40% of cases in LATAM and 53% and 51% of cases in Europe, respectively. Tumors from Europe were more commonly located in the proximal stomach (cardia/gastroesophageal junction), whereas those from LATAM were predominantly found in the middle region (body/fundus). High-grade tumors were significantly more prevalent in LATAM (51%) compared to Europe (23%). Additionally, LATAM tumors exhibited a higher mean percentage of the signet ring cell (SRC) component. In LATAM, 52 cases (38%) showed at least 1% SRC, with a mean SRC percentage of 39.7% among cases with any SRC component. In contrast, 33 cases (27%) from Europe displayed at least 1% SRC, with a mean percentage of 30.2% among cases with SRC components (Table 1, additional data not shown).

The European cohort exhibited a higher frequency of HER2 positivity compared to the LATAM cohort (11% vs. 4%, *p* = 0.048). Mismatch repair (MMR) deficiency was slightly more common in cases from Europe (15% vs. 10%), as was EBV expression (7% vs. 3%). However, these differences were not statistically significant (Table 1).

**Fig. 1:**
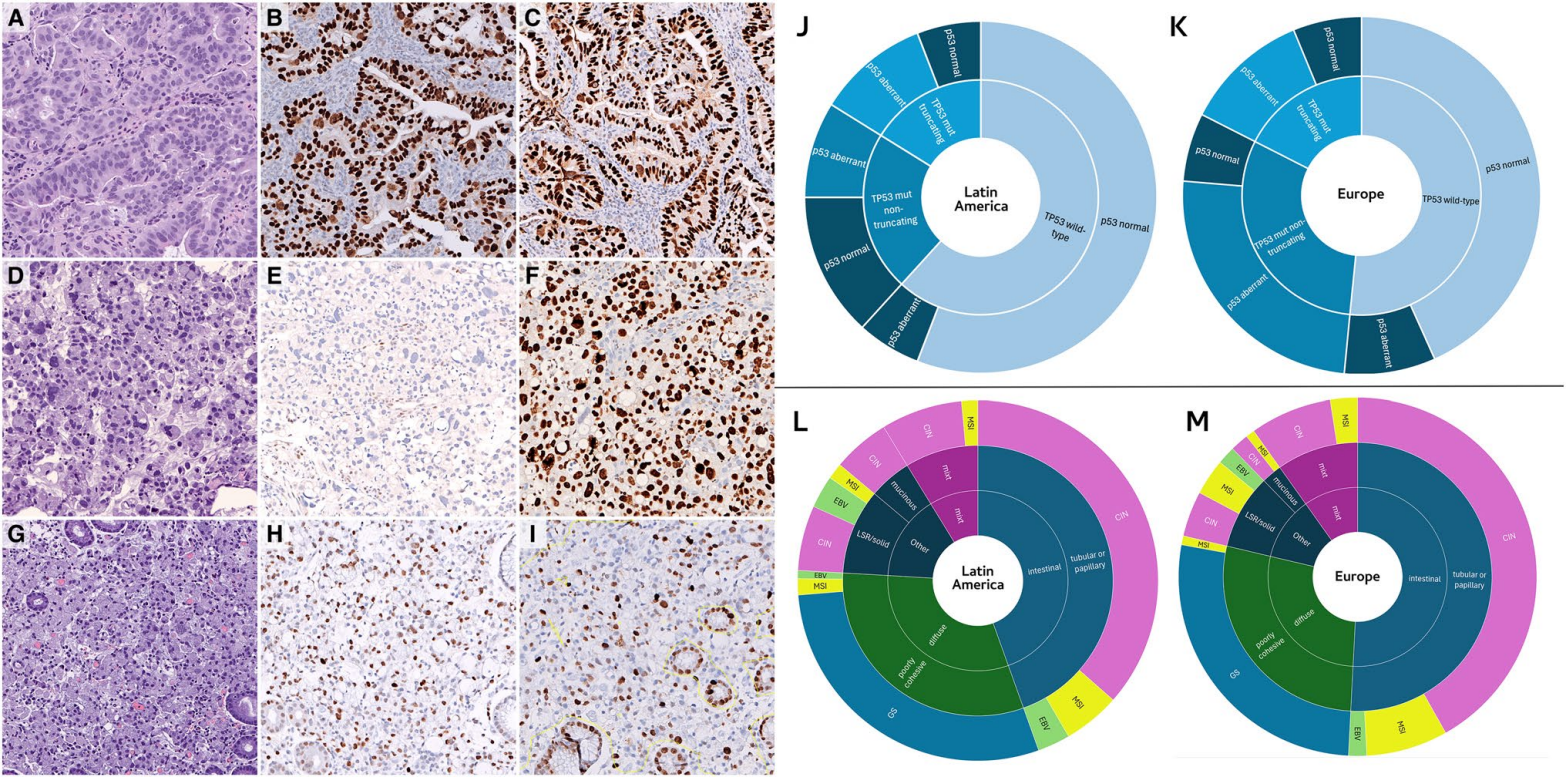
**A–I** Immunohistochemical patterns of p53 and Ki67 expression. A tubular low-grade adenocarcinoma (HE, 20X, A) shows strong p53 immunostaining in all tumor cells (aberrant pattern, H-score = 300) (p53, 20X, B) and high proliferative activity (Ki67, 20X, C). An undifferentiated carcinoma (HE, 20X, D) exhibits a complete absence of p53 immunostaining in all tumor cells (aberrant pattern, H-score = 0) (p53, 20X, E) and high proliferative activity (Ki67, 20X, F). A poorly cohesive adenocarcinoma with signet ring cell morphology (HE, 20X, G) presents heterogeneous p53 expression in some tumor cells (normal pattern, H-score = 185) (p53, 20X, H) and proliferative activity below the median (Ki67, 20X, I). **J–K:** Distribution of cases according with TP53 mutational status (inner ring) and p53 immunohistochemical pattern (external ring) in Latin America (J) and Europe (K). **L, M** Correlation between histological Lauren’s classification (inner ring), WHO classification (middle ring) and the molecular surrogate immunohistochemical classification (external ring) in Latin America (L) and Europe (M)

Of all cases, 35% (*n* = 89) exhibited a p53 aberrant pattern of immunohistochemical expression, with a significantly higher frequency in the European cohort (46% *vs* 26%, *p* = 0.001; Table 1). Out of 165 cases suitable for both *TP53* mutational analysis and p53 immunohistochemical evaluation, 75 (45%) were confirmed to harbor a mutation. Of these, 45 (60%) were non-truncating mutations, while 30 (40%) were truncating mutations. (Table 2). The classification based on the dichotomous immunohistochemical pattern (aberrant vs normal pattern) was associated with the *TP53* mutational status (*p* < 0.001). Using this approach, 67% (*n* = 50) of *TP53* mutated cases and 87% (*n* = 78) of the *TP53* wild-type cases were accurately classified by immunohistochemistry (Table 2). The sensitivity of the IHC classification for detecting TP53 truncating and non-truncating mutations was consistent, with both types showing a detection rate of 67%. However, among cases with aberrant p53 expression, the staining pattern was significantly associated with the mutation type (*p* < 0.001). All cases with non-truncating mutations exhibited pronounced p53 overexpression, with H-scores ranging from 280 to 300. In contrast, 18 (90%) of the 20 cases with truncating mutations demonstrated a complete loss of p53 protein expression, reflected in an H-score of 0. Although, the p53 IHC aberrant pattern was more frequently encountered in tumors from Europe, there were no significant differences regarding the *TP53* mutational status or type (truncating vs non-truncating) between the two regions. Nevertheless, both *TP53* mutations and p53 overexpression were significantly associated with the intestinal histological type (65% and 64%, respectively), higher proliferative activity and lower SRC content (Fig. 1A–K, Supplementary Tables 1 and 2). No specific relationship between *TP53* mutational status or p53 IHC expression and CD3, CD8 or PD-L1 was observed (data not shown). Figure 1A–I displays immunohistochemical patterns of p53 and Ki-67 expression in three cases.

**Table 2:** Correlation between *TP53* mutational *status* and p53 immunohistochemical expression pattern

	Total, *n* = 165	*TP53* mutational status			
		WT, *N* = 90 (55%)	Non-truncating mutation, *n* = 45 (27%)	Truncating mutation, *N* = 30 (18%)	*p* value
P53 H-score					
Mean (mean SD)	161.7 (105.7)	141 (82.4)	258.7 (74.2)	64.9 (82.4)	***0****.****000***
Median (Min–Max)	160 (0–300)	137.5 (0–300)	290 (16–300)	137.5 (0–102)	
P53 IHC pattern					
Aberrant pattern	62 (38%)	12 (13%)	30 (67%)	20 (67%)	***0****.****000***
Normal pattern	103 (62%)	78 (87%)	15 (33%)	10 (33%)	

Values in bold indicate statistically significant differences (*p* < 0.05), while italicized values represent non-significant trends

GC cases from Europe showed higher frequency of PD-L1 expression, regardless of the CPS cut-off point (*p* < 0.001 for CPS ≥ 1, ≥ 5, and ≥ 10) (Table 1). The European cases were also enriched in FoxP3-positive tumor-infiltrating lymphocytes (< 0.001). Across the entire series, PD-L1 CPS was significantly correlated with the content of CD3-positive (*p* < 0.001 for CPS ≥ 5, and ≥ 10), CD8-positive (*p* < 0.001 for CPS ≥ 5, and ≥ 10) and FOXP3-positive cells (*p* < 0.001 for CPS ≥ 5 and *p* < 0.002 for CPS ≥ 10). These association correlates with the differences in FOXP3 distribution observed between Europe and LATAM. However, no regional differences were detected in the distribution of CD3 or CD8-positive cells (Table 1). Additionally, in cases from Europe a significantly higher proliferative index was observed (mean Ki67: 75.8% *vs* 69.0%, *p* = 0.02) (Table 1).

Following the molecular classification by the immunohistochemical algorithm (Table 1, Fig. 1L, M) the most striking differences concerned that cases classified as CIN tumors were underrepresented in the LATAM cohort (21% in LATAM vs 43% in Europe) while the GS group was the most frequent genomic subtype in LATAM countries (62%). By categorizing cases into two molecular groups based on their immunogenicity (immune: MSI/EBV vs. non-immune: GS/CIN), cases classified within the immune subtype demonstrated significant associations with distinct features, including lymphoid-rich stroma/solid morphological phenotype, higher frequency of PD-L1 expression, increased proliferative activity, reduced signet-ring cell (SRC) component, and elevated levels of CD8-positive immune cells (Supplementary Table 3). When focusing only on the non-immune subgroup, regional differences in PD-L1 expression between Europe and LATAM were still evident (*p* < 0.001 for CPS ≥ 1, CPS ≥ 5 and ≥ 10).

**Fig. 2:**
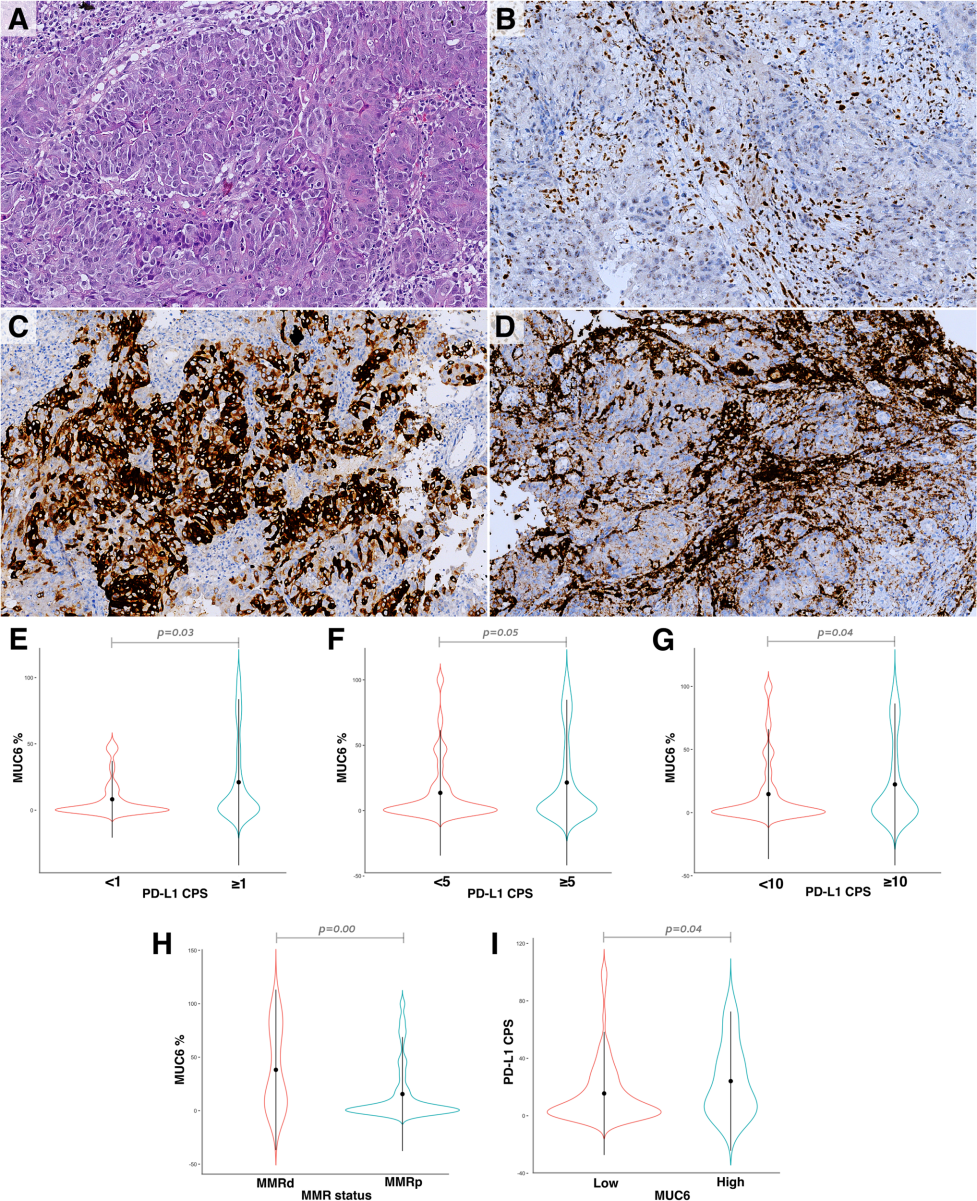
**A–D** A high-grade tubular adenocarcinoma (HE, 10X, A) shows loss of MLH1 and PMS2 nuclear expression in all tumor cells (MLH1, 20X, B). There is intense immunostaining of MUC6 in most neoplastic cells (MUC6, 20X, C) and diffuse expression of PD-L1 in the inflammatory component with a CPS score of 75 (PD-L1, 20X, D). **E–G** Correlation between the percentage of tumor cells expressing MUC6 and PD-L1 CPS categories: CPS ≥ 1 (E), CPS ≥ 5 (F), and CPS ≥ 10 (G). **H** Variation in MUC6 expression in tumor cells based on MMRp status. **I** Differences in PD-L1 CPS scores stratified by MUC6 expression levels (high: > 50% of tumor cells; low: ≤ 50% of tumor cells)

The mean percentage of positive cells for MUC6 was significantly higher in cases from EU compared to those from LATAM (25.6% *vs* 12.4%, *p* < 0.001; Table 1). High MUC6 expression was observed in 26% (*n* = 67) of cases and demonstrated a significant association with PD-L1 positivity across all CPS thresholds (*p* = 0.03, 0.05, and 0.04 for CPS ≥ 1, ≥ 5, and ≥ 10, respectively) (Table 3, Fig. 2A–G). Approximately 48% of MMRd cases exhibited MUC6 overexpression, in contrast to only 23% of MMRp cases (*p* = 0.003) (Fig. 2H). When focusing only within the non-immune GS/CIN subtypes, this correlation between MUC6 and PD-L1 clinically relevant cut-off point was missed. However, we observed a slightly but statistically significant overexpression of PD-L1 in cases presenting MUC6 staining in at least 50% of tumor cells (Fig. 2I).

**Table 3:** Clinicopathological variables according to MUC6 immunohistochemical expression

	Total, *n* = 258	MUC6 immunohistochemical expression		
		Neg/Low, *n* = 191 (74%)	High, *n* = 67 (26%)	*p*-value
Region				
Europe	121 (47%)	74 (39%)	47 (70%)	** < *0****.****001***
LATAM	137 (53%)	117 (61%)	20 (30%)	
MMR status				
MMRp	225 (88%)	173 (92%)	52 (78%)	***0****.****003***
MMRd	31 (12%)	16 (8%)	15 (22%)	
PD-L1 CPS				
< 1%	47 (20%)	41 (23%)	6 (10%)	***0****.****03***
> = 1%	191 (80%)	137 (77%)	54 (90%)	
< 5%	89 (37%)	73 (41%)	16 (27%)	** < *0****.****05***
> = 5%	149 (63%)	105 (59%)	44 (73%)	
< 10%	118 (50%)	95 (53%)	23 (38%)	***0****.****04***
> = 10%	120 (50%)	83 (47%)	37 (62%)	

Values in bold indicate statistically significant differences (*p* < 0.05), while italicized values represent non-significant trends

*Helicobacter pylori* was successfully assessed in 163 cases and was detected in more than 80% of GC cases from both regions (89% in LATAM and 85% in Europe). There were no statistically significant differences regarding *H. pylori* prevalence or abundance in tumors from LATAM and Europe (Supplementary Table 4). Furthermore, no significant relationships were observed between *H. pylori* infection and histological type, tumor location, expression of GC predictive biomarkers (HER2, MMRd or EBV infection) or the number of CD3, CD8, PD-L1 or FoxP3 positive cells (data not shown).

## Discussion

Our study demonstrates that there are significant biological differences in gastroesophageal cancer between Europe and LATAM. These differences are centered on cell-cycle and molecular drivers expressed by tumor cells (HER2, p53, and Ki67), phenotypic attributes (high differentiation grade, SRC content, and MUC6 expression), and microenvironment features (FOXP3 and PD-L1).

While the frequency of diffuse/poorly cohesive adenocarcinoma was very similar between the two cohorts, we observed a slight predominance of SRC content in tumors from LATAM. This high SRC content has been previously described in LATAM series in association with young age; however, studies on the prognostic effect of this finding remain contradictory [26, 27]. Additionally, a predominance of SRC carcinoma has been noted in the LATAM population in a study conducted in the United States, suggesting a potential role of genetic factors in the development of these tumors [27]. The same authors performed a specific analysis of the Hispanic population in the SEER (National Cancer Institute’s Surveillance, Epidemiology, and End Results) database obtaining similar results.

Consensus recommendations and guidelines for GEJ cancer management from Europe and North America estimate HER2 overexpression between 10 and 20%, and 12% and 23%, respectively [28, 29]. HER2 was amplified in 17% of cases in the TCGA (The Cancer Genome Atlas) Research Network’s molecular classification of GEJ cancer [30]. Remarkably, our findings reveal a significantly lower rate of HER2 positivity in LATAM (4%), underscoring a notable regional difference that could have profound implications for targeted therapies and patient outcomes. In Costa Rica and Mexico a similar low prevalence of HER2 overexpression was described [26, 31, 32], whereas in Chile the prevalence was found to be higher [33, 34]. One possible explanation for the difference in HER2 frequency observed in both settings may be the deviation in the distribution of molecular subtypes in LATAM compared to the TCGA classification report with significant enrichment in GS cases and a relatively low frequency of CIN cases in LATAM. *TP53* mutations are enriched in the chromosomal instability (CIN) molecular subtype, which concentrates most HER2-amplified cases (71% of CIN cases were *TP53* mutated, according to TCGA) [30]. Additionally, the molecular classification from the Asian Cancer Research Group (ACRG) indicates that copy number variations (CNVs) in various genes, including HER2, are frequently encountered in the MSS/*TP53* inactive group, where *TP53* mutations are more prevalent [34]. In our series, *TP53* mutations were significantly correlated with other features characteristic of the CIN subtype (intestinal histology, HER2 amplification) and the frequency of *TP53* mutations in Latin America was slightly lower (40%) compared to Europe (49%). The previously discussed enrichment of SRC in Latin countries may also be a consequence of the overrepresentation of the GS molecular subtype in this population. Additionally, we observed a significant increase in SRC levels in TP53 wild-type cases and in those displaying a p53 normal immunohistochemical pattern, as previously noted in a Chilean cohort [33].

Notably, the immunohistochemical analysis of p53 revealed an aberrant pattern of expression in nearly half (46%) of the European cases, a prominent feature compared to LATAM cases (26%). This dichotomous classification of p53 immunohistochemical expression showed good specificity for predicting the *TP53* mutational status (87%), as most *TP53* wild-type cases were accurately classified as having a normal pattern of protein expression. However, the sensitivity of this approach was modest (65%), with *TP53* mutational status being adequately predicted in only two-thirds of the cases classified as having a p53 aberrant pattern by immunohistochemistry. One limitation of this method is the intratumoral heterogeneity of the protein, which has been demonstrated in gastric and GEJ adenocarcinoma and may explain the decreased sensitivity when applied to small biopsies [36]. Despite this limitation, in our series, p53 immunohistochemical aberrant expression was associated with characteristics typical of CIN tumors (intestinal histology and HER2 overexpression), supporting the utility of p53 immunohistochemistry in identifying this more aggressive subtype [36, 37]. Furthermore, the proliferative Ki-67 index, another differential characteristic between European and LATAM tumors, was consistently associated with both the presence of *TP53* mutations and p53 aberrant pattern of protein expression, aligning with our hypothesis.

An interesting finding in our study is the differential expression profile of MUC6 between Europe and LATAM. MUC6 is a gel-forming mucin constitutively expressed in the stomach, pancreas, duodenum, and female reproductive tract [38]. In gastric mucosa, MUC6 has been identified as an oncogenic driver, mutated in around 20% of all cases [35, 39], and its downregulation associated with tumor progression [40]. Notably, we found a significant positive relationship between PD-L1 and MUC6 expression, irrespective of the CPS cut-off point. It has been suggested that mucins may play a role in immune regulation in solid tumors through various mechanisms, including direct interaction with dendritic cells, macrophages, and natural killer cells; interaction with Toll-like receptors; the generation of neoantigens; or their transcriptional regulation by pro-inflammatory cytokines [41–43]. We identified a significant association between MUC6 expression and MMRd, a relationship previously reported in colorectal carcinoma [44]. In our series, the strong correlation between PD-L1 and MUC6 was primarily driven by the immune-active MSI group. However, within the non-immune GS/CIN subtypes, a subtle correlation between these markers persisted, highlighting a potentially nuanced interplay that warrants further investigation. This is especially important in the era of immunotherapy for gastric cancer, where significant scientific effort is focused on understanding the mechanisms regulating the immune response and refining biomarkers for patient selection. Additionally, PD-L1 expression was significantly higher in Europe, and a low prevalence of PD-L1 expression was previously observed in an exploratory analysis conducted in Chile [34].

Our analysis identified significant distinctions between immune-related tumors (EBV/MSI) and non-immune tumors (GS/CIN) across multiple dimensions, including morphology, PD-L1 expression, proliferative activity, SRC component, and the presence of CD8-positive immune cells. These differences may hold functional relevance. Nevertheless, despite these findings, the distribution of immune and non-immune subtypes was comparable between LATAM and European cohorts. This similarity suggests that other intrinsic factors may be responsible for the observed regional differences.

Finally, our differential analysis of *H. pylori* prevalence and abundance revealed no differences between LATAM and Europe. Therefore, at least in our study, none of the observed regional differences can be attributed to *H. pylori* infection.

We conducted a detailed morphological and protein expression analysis using IHC and ISH to explore intrinsic differences between Latin American and European gastric cancer (GC) cohorts. To achieve this, we utilized a molecular surrogate classification based on morphology and IHC, a method that has demonstrated both clinical value and feasibility [20]. However, it is important to recognize the limitations of this approach. The surrogate classification may not fully align with the more intricate TCGA molecular classification, which integrates multi-omics data for a comprehensive analysis. This highlights the need for further studies to refine and validate surrogate methods in diverse populations.

Another limitation of our study is that all analyses were conducted on endoscopically obtained biopsies, which reflect only the superficial portion of the tumor. This limitation is particularly relevant in GEJ cancer, where tumor heterogeneity is a prominent characteristic. Additionally, our study lacks data on Claudin-18.2 and FGFR2b expression—key targets in emerging antibody–drug therapies—further constraining its scope. Finally, it is important to consider that for PD-L1 analysis we have utilized the 22C3 antibody–based LDT on the Ventana BenchMark platform. While this may be considered a limitation, published harmonization studies have shown comparable results when compared with the regulatory-approved, PD-L1 IHC 22C3 pharmDx test, supporting the validity of our approach [45].

In summary, the LEGACy study underscored significant biological distinctions between European and LATAM patients with gastric and GEJ cancer:

1. Distribution of molecular subtypes: our results suggest a deviation from TCGA results, notably showing a decreased proportion of CIN and HER2-positive cases, and an overrepresentation of GS tumors, in LATAM. The increased SRC content observed in LATAM cases may align with this trend.
2. *TP53* mutations: While the immunohistochemical approach for predicting *TP53* mutations has limitations, we did find a significant correlation between p53 aberrant expression and other CIN features: intestinal histology and HER2 overexpression.
3. Correlation between MUC6 and PD-L1: Both MUC6 and PD-L1 overexpression was seen more frequently in European cases. Across both cohorts, MMRd cases showed high expression levels of MUC6 and PD-L1. MMRp cases displayed a weaker but still significant association between these two markers. This lends support the hypothesis of MUC6 acting as an immune regulator, which suggests avenues for further investigation.
4. *H. pylori* infection relevance: differences in *H. pylori* distribution or abundance were not observed in the comparison between European and LATAM cases; hence the observed differential features cannot be attributed to this infection.

Altogether, the findings of this study underscore a need for region-specific approaches in gastric and GEJ cancer treatment. In LATAM, where tumors exhibit lower HER2 and PD-L1 expression, current treatment strategies may be less effective. Further research tailored to the unique biological profiles in different regions is crucial for addressing these disparities effectively.

### Centers

ANAX: Anaxomics SL (Spain).

ECPC: European Cancer Patient Coalition (Belgium).

GENPAT: Genpat SL (Paraguay).

IAF: Institute Alexandre Fleming (Argentina).

INCAN: National Cancer Institute (Mexico).

INCLIVA: Biomedical Research Center INCLIVA (Spain).

IPATIMUP: Institute of Molecular Pathology and Immunology of the University of Porto (Portugal).

PUC: Pontificia Universidad Católica de Chile (Chile).

ULEI: University of Leipzig (Germany).

VHIO: Valld’HebrónInstitut of Oncology (Spain).

VUMC: Amsterdam UMC location VU University Medical Center (Netherlands).

## Supplementary Information

Below is the link to the electronic supplementary material.

Supplementary file1 (DOCX 16 KB)

Supplementary file2 (DOCX 16 KB)

Supplementary file3 (DOCX 19 KB)

Supplementary file4 (DOCX 16 KB)

Supplementary file5 (DOCX 24 KB)
