# Supplementary material for: Comprehensive histopathological analysis of gastric cancer in European and Latin America populations reveals differences in PDL1, HER2, p53 and MUC6 expression
Source: Gastric Cancer. 2025 Jan 5;28(2):160–73. doi: 10.1007/s10120-024-01578-3 (PMC11842524; doi:10.1007/s10120-024-01578-3)
Supplement: Supplementary file 1 — Supplementary file1 (DOCX 16 KB) [file 10120_2024_1578_MOESM1_ESM.docx]

Supplementary table 1. Clinicopathological variables according to *TP53* mutational status

|  |  | ***TP53* mutational status** | |  |
| --- | --- | --- | --- | --- |
|  | ***Total***  n=168 | **WT**  n=92 (55%) | **Mutated**  n=76 (45%) | ***P value*** |
| **Laurén Classification** |  |  |  |  |
| Intestinal | 88 (53%) | 39 (42%) | 49 (65%) | ***0.009*** |
| Diffuse | 41 (24%) | 30 (32%) | 11 (14%) |  |
| Mixed | 17 (10%) | 8 (9%) | 9 (12%) |  |
| Unclassifiable | 22 (13%) | 15 (16%) | 7 (9%) |  |
| **Signet ring cell (SRC) content** |  |  |  |  |
| Mean (mean SD) | 11.3 (25.8) | 16.2 (30.4) | 5.3 (17.1) | ***0.006*** |
| Median (Min-Max) | 0 (0-100) | 0 (0-100) | 0 (0-100) | ***0.004*** |
| **EBV infection** |  |  |  | ***0.010*** |
| Negative | 157 (93%) | 82 (89%) | 75 (99%) |  |
| Positive | 11 (7%) | 10 (11%) | 1 (1%) |  |
| **HER2** |  |  |  |  |
| Negative | 151 (91%) | 90 (98%) | 61 (82%) | ***0.001*** |
| Positive | 15 (9%) | 2 (2%) | 13 (18%) |  |
| **Ki-67 activity** |  |  |  |  |
| Mean (mean SD) | 75.4 (22.1) | 69.4 (24.8) | 82.7 (15.6) | ***<0.001*** |
| Median (Min-Max) | 83 (5.4-100) | 77 (5.4-100) | 86 (23-100) | ***<0.001*** |
