# Supplementary material for: Comprehensive histopathological analysis of gastric cancer in European and Latin America populations reveals differences in PDL1, HER2, p53 and MUC6 expression
Source: Gastric Cancer. 2025 Jan 5;28(2):160–73. doi: 10.1007/s10120-024-01578-3 (PMC11842524; doi:10.1007/s10120-024-01578-3)
Supplement: Supplementary file 2 — Supplementary file2 (DOCX 16 KB) [file 10120_2024_1578_MOESM2_ESM.docx]

Supplementary table 2. Clinicopathological variables according to p53 immunohistochemical expression

|  |  | **p53 IHC pattern** | |  |
| --- | --- | --- | --- | --- |
|  | ***Total***  n=253 | **Aberrant pattern**  n=164 (65%) | **Normal pattern**  n=89 (35%) | ***P value*** |
| **Laurén Classification** |  |  |  |  |
| Intestinal | 124 (49%) | 67 (41%) | 57 (64%) | ***0.003*** |
| Diffuse | 73 (29%) | 56 (34%) | 17 (19%) |  |
| Mixed | 23 (9%) | 15 (9%) | 8 (9%) |  |
| Unclassifiable | 33 (13%) | 26 (16%) | 7 (8%) |  |
| **Signet ring cell (SRC) content** |  |  |  |  |
| Mean (mean SD) | 12.0 (26.2) | 14.3 (28.4) | 6.8 (21.3) | ***0.030*** |
| Median (Min-Max) | 0 (0-100) | 0 (0-100) | 0 (0-100) | ***0.005*** |
| **EBV infection** |  |  |  |  |
| Negative | 239 (95%) | 153 (93%) | 86 (98%) | *0.129* |
| Positive | 13 (5%) | 11 (7%) | 2 (2%) |  |
| **HER2** |  |  |  |  |
| Negative | 236 (93%) | 154 (95%) | 76 (87%) | ***0.028*** |
| Positive | 19 (7%) | 8 (5%) | 11 (13%) |  |
| **FoxP3** |  |  |  |  |
| Mean (mean SD) | 93.0 (94.0) | 74.0 (86.7) | 126.5 (97.3) | ***<0.001*** |
| Median (Min-Max) | 69 (0.4-564) | 50.5 (0.4-564) | 98 (3.3-450) | ***<0.001*** |
| **Ki-67 activity** |  |  |  |  |
| Mean (mean SD) | 72.7 (23.7) | 68.3 (25.2) | 80.5 (18.4) | ***<0.001*** |
| Median (Min-Max) | 80 (4-100) | 77 (4-100) | 85 (20-100) | ***<0.001*** |
