# Supplementary material for: Comprehensive histopathological analysis of gastric cancer in European and Latin America populations reveals differences in PDL1, HER2, p53 and MUC6 expression
Source: Gastric Cancer. 2025 Jan 5;28(2):160–73. doi: 10.1007/s10120-024-01578-3 (PMC11842524; doi:10.1007/s10120-024-01578-3)
Supplement: Supplementary file 3 — Supplementary file3 (DOCX 19 KB) [file 10120_2024_1578_MOESM3_ESM.docx]

Supplementary table 3. Clinicopathological variables according to the molecular immune vs non-immune subtype

|  |  | **Molecular subtype** | |  |
| --- | --- | --- | --- | --- |
|  | ***Total***  n=259 | **Immune (MSI / EBV)**  n= 44 (17%) | **Non-immune (GS / CIN)**  n= 215 (83%) | ***P value*** |
| **Region** |  |  |  |  |
| LATAM | 137 (53%) | 22 (50%) | 115 (53%) | *0.672* |
| Europe | 122 (47%) | 22 (50%) | 100 (47%) |  |
| **Laurén Classification** |  |  |  |  |
| Intestinal | 124 (48%) | 22 (50%) | 101 (47%) | ***0.000*** |
| Diffuse | 77 (30%) | 4 (9%) | 73 (34%) |  |
| Mixed | 24 (9%) | 5 (11%) | 19 (9%) |  |
| Unclassifiable | 35 (13%) | 13 (30%) | 22 (10%) |  |
| **WHO classification** |  |  |  |  |
| Tubular/papillary | 121 (47%) | 22 (50%) | 101 (47%) | ***0.000*** |
| Poorly cohesive | 77 (30%) | 4 (9%) | 73 (34%) |  |
| Mucinous | 12 (4%) | 1 (2%) | 9 (4%) |  |
| Mixed | 22 (9%) | 5 (11%) | 19 (9%) |  |
| Lymphoid stroma-rich/solid | 26 (10%) | 12 (27%) | 13 (6%) |  |
| **PD-L1 CPS** |  |  |  |  |
| <1% | 47 (20%) | 5 (22%) | 42 (20%) | *0.121* |
| >=1% | 191 (80%) | 39 (78%) | 152 (80%) |  |
| <5% | 89 (37%) | 9 (20%) | 80 (41%) | ***0.010*** |
| >=5% | 149 (63%) | 35 (80%) | 114 (59%) |  |
| <10% | 118 (50%) | 15 (34%) | 103 (53%) | ***0.022*** |
| >=10% | 120 (50%) | 29 (66%) | 91 (47%) |  |
| **CD8** |  |  |  |  |
| Mean (mean SD) | 7771 (985) | 1157 (1280) | 686 (889) | ***0.018*** |
| Median (Min-Max) | 504 (5-9999) | 976 (19-7069) | 486 (5-9999) |  |
| **Signet ring cell (SRC) content** |  |  |  |  |
| Mean (mean SD) | 11.9 (26.1) | 1.3 (4.8) | 14.1 (28.1) | ***0.001*** |
| Median (Min-Max) | 0 (0-100) | 0 (0-25) | 0 (0-100) |  |
| **Ki-67 activity** |  |  |  |  |
| Mean (mean SD) | 72.2 (23.8) | 85 (15.5) | 69.9 (24.1) | ***0.000*** |
| Median (Min-Max) | 80 (4-100) | 50 (21.6-100) | 77.8 (4-100) |  |
