# Supplementary material for: Comprehensive histopathological analysis of gastric cancer in European and Latin America populations reveals differences in PDL1, HER2, p53 and MUC6 expression
Source: Gastric Cancer. 2025 Jan 5;28(2):160–73. doi: 10.1007/s10120-024-01578-3 (PMC11842524; doi:10.1007/s10120-024-01578-3)
Supplement: Supplementary file 4 — Supplementary file4 (DOCX 16 KB) [file 10120_2024_1578_MOESM4_ESM.docx]

Supplementary table 4. *Helicobacter pylori* infection and abundance in European

*vs* Latin American (LATAM) countries

|  | ***Total***  n=163 | **LATAM**  n=62 (38%) | **Europe**  n=101 (62%) | ***P value*** |
| --- | --- | --- | --- | --- |
| ***H. pylori*** |  |  |  |  |
| Absent | 22 (13%) | 7 (11%) | 15 (15%) |  |
| Present | 141 (87%) | 55 (89%) | 86 (85%) | *0.518* |
| ***H. pylori* abundance** |  |  |  |  |
| Mean (mean SD) | 1.67 (1.51) | 1.84 (1.55) | 1.56 (1.48) | *0.242* |
| Median (Min-Max) | 1.04 (0-6.07) | 1.32 (0-5.7) | 0.96 (0-6.1) | *0.214* |
