# Supplementary material for: Comprehensive histopathological analysis of gastric cancer in European and Latin America populations reveals differences in PDL1, HER2, p53 and MUC6 expression
Source: Gastric Cancer. 2025 Jan 5;28(2):160–73. doi: 10.1007/s10120-024-01578-3 (PMC11842524; doi:10.1007/s10120-024-01578-3)
Supplement: Supplementary file 5 — Supplementary file5 (DOCX 24 KB) [file 10120_2024_1578_MOESM5_ESM.docx]

**Supplementary table 5: Study sites and study members**

| Amsterdam UMC, Department of Medical Oncology, Cancer Center Amsterdam, Amsterdam, The Netherlands and Oncode Institute | De boelelaan, 1117  1081 HV Amsterdam, The Netherlands | Tessa Suzanne van Schooten |
| --- | --- | --- |
|  |  | Sarah Derks |
|  |  | Roos Pouw |
| Instituto Investigación Sanitaria INCLIVA (INCLIVA), Medical OncologyDepartment, Hospital Clínico Universitario de Valencia. Pathology Department, Bioestatistic Unit INCLIVA, Precision Unit Department, Gastroenterology Department. | Avenida Blasco Ibáñez, 17, 46010, Valencia, Spain | Tania Fleitas Kanonnikoff |
|  |  | Andrés Cervantes Ruipérez |
|  |  | Elena Jiménez-Martí |
|  |  | Ana Miralles-Marco |
|  |  | Carolina Martínez-Ciarpaglini |
|  |  | Juan A. Carbonell Asins |
|  |  | Lorena Alarcón-Molero |
|  |  | Manuel Cabeza Segura |
|  |  | Beatriz Lopez |
|  |  | Sergio Romero |
|  |  | Marisol Huerta |
|  |  | Andrea Marín-Alonso |
|  |  | Pilar Rentero |
|  |  | Valentina Gambardella |
|  |  | Andrés Peña |
|  |  | Pablo Navarro |
| Institute of Molecular Pathology and Immunology of the University of Porto (IPATIMUP). Department of Anatomic Pathology, Centro Hospitalar São João | Rua Júlio Amaral de Carvalho 45  4200-135 Porto, Portugal | Fatima Carneiro |
|  |  | Ceu Figueiredo |
|  |  | Rita Barros |
|  |  | Rui Ferreira |
|  |  | Joana Pereira-Marques |
|  |  | Melissa Mendes-Rocha |
|  |  | João Antunes |
|  |  | Andreia Costa |
|  |  | Rosa Maria Azevedo |
| Instituto Nacional de Cancerología (INCAN), Translational Medicine Laboratory & GI Cancer Department | San Fernando N.22, Colonia Seccion XVI,  14080, Mexico City, Mexico | Erika Ruiz-García |
|  |  | Edith A. Fernández-Figueroa |
|  |  | Alberto Leon-Takahashi |
|  |  | Nayeli Ortiz-Olvera |
|  |  | Nadia Melissa Valdez-Reyes |
|  |  | Roberto Herrera-Goepfert |
|  |  | Jesús Argueta-Donohué |
|  |  | Juan Carlos Falcón-Martínez |
|  |  | Pilar Salas Rodríguez |
|  |  | Saul Lino-Silva |
|  |  | Angelica Hernandez-Guerrero |
|  |  | Consuelo Diaz-Romero |
|  |  | Abelardo Meneses-García |
| Valld’Hebron Institute of Oncology (VHIO), Medical Oncology Department, Data Science Department, Cancer Genomics Lab. | Valld’Hebron University Hospital  Centro Cellex, Calle Natzaret, 115-117  08035 Barcelona, Spain | Maria Alsina |
|  |  | Marc Diez |
|  |  | Rodrigo Dienstmann |
|  |  | Fiorella Ruiz-Pace |
|  |  | Ana Vivancos |
|  |  | Judit Matito |
|  |  | Agatha Martín |
|  |  | Marina Gomez |
|  |  | Ester Castilla |
|  |  | María Vila |
| Pontificia Universidad Católica de Chile, Medical Oncology Department, Gastroenterology department, Pathology department, Millennium Institute for Immunology and Immunotherapy, Center for Prevention. | Diagonal Paraguay 362  8330077, Santiago,Chile | Marcelo Garrido |
|  |  | Arnoldo Riquelme |
|  |  | Mauricio Pinto |
|  |  | Matias Muñoz |
|  |  | Maria Loreto Bravo |
|  |  | Miguel Cordova |
|  |  | Valentina Ortiz |
|  |  | Margarita Pizarro |
|  |  | Gareth Owen |
|  |  | Gonzalo Latorre |
|  |  | Juan Carlos Roa |
|  |  | Erick Riquelme |
| GenPat | Guido Spano 1448 e/ Ohiggins y Dr. Morra, Asunción, Paraguay | Carmelo Caballero |
|  |  | Hugo Boggino |
| Instituto de Previsión Social, Medical Oncology Department, Gastroenterology Department, Pathology Department | Constitución esq. Luis A. de Herrera, Asunción, Paraguay | Eva Lezcano |
|  |  | Daniel Cantero |
|  |  | Cinthia Gauna |
|  |  | Horacio Lezcano |
|  |  | Maria Rita Pereira |
|  |  | Mariela Romero |
|  |  | Loida Caballero |
| Instituto Alexander Fleming,  Medical Oncology Department | Avenida Crámer, 1180C1426ANZ Buenos Aires, Argentina | Federico Esteso |
|  |  | Juan Manuel O’Connor |
|  |  | Romina Luca |
|  |  | Berenice Freile |
|  |  | Luis Caro, gastroenterologist |
|  |  | Mora Amat, pathologist |
| AnaxomicsBiotec | AnaxomicsBiotech, S.L. C/ Diputació 237, 1-1, 08007 Barcelona, Spain | Judith Farrés |
|  |  | José Manuel Mas |
|  |  | José Luís Ruiz |
|  |  | Hugo Somoza |
| University Cancer Center Leipzig (UCCL), Leipzig University Medical Center.Department of Oncology, Gastroenterology, Hepatology and Pulmonology. | Liebigstraße 22, 04103 Leipzig, Germany | Florian Lordick |
|  |  | Jeannette Vogt |
| European Cancer Patient Coallition, Direction& EU Affairs Dpt. | Avenue des Arts 6, B-1210, Brussels, Belgium | Antonella Cardone |
|  |  | Charis Girvalaki |
|  |  | Klevisa Ceka |
|  |  | Alexander Rodriguez |
|  |  | Paulina Gono |
|  |  | Aina Errando |
|  |  | Ane de las Heras |
|  |  | Nicola Di Flora |
